# Changes in Semi-Arid Plant Species Associations along a Livestock Grazing Gradient

**DOI:** 10.1371/journal.pone.0040551

**Published:** 2012-07-09

**Authors:** Hugo Saiz, Concepción L. Alados

**Affiliations:** Grupo de Conservación de la biodiversidad, Departamento de Biodiversidad y Restauración, Instituto Pirenaico de Ecología, Consejo Superior de Investigaciones Científicas, Zaragoza, Spain; USDA-ARS, United States of America

## Abstract

In semi-arid ecosystems, vegetation is heterogeneously distributed, with plant species often associating in patches. These associations between species are not constant, but depend on the particular response of each species to environmental factors. Here, we investigated how plant species associations change in response to livestock grazing in a semi-arid ecosystem, Cabo de Gata-Níjar Natural Park in South East Spain. We established linear point-intercept transects at four sites with different grazing intensity, and recorded all species at each point. We investigated plant associations by comparing the number of times that each pair of species occurred at the same spatial point (co-occurrences), with the expected number of times based on species abundances. We also assessed associations for each shrub and grass species by considering all their pairs of associations and for the whole plant community by considering all pairs of associations on each site. At all sites, the plant community had a negative pattern of association, with fewer co-occurrences than expected. Negative association in the plant community increased at maximum grazing intensity. Most species associated as expected, particularly grass species, and positive associations were most important at intermediate grazing intensities. No species changed its type of association along the grazing gradient. We conclude that in the present plant community, grazing-resistant species compete among themselves and segregate in space. Some shrub species act as refuges for grazing-sensitive species that benefit from being spatially associated with shrub species, particularly at intermediate grazing intensities where positive associations were highest. At high grazing intensity, these shrubs can no longer persist and positive associations decrease due to the disappearance of refuges. Spatial associations between plant species and their response to grazing help identify the factors that organize plant communities, and may contribute to improving management of semi-arid ecosystems.

## Introduction

Plant species associations are a fundamental aspect of plant community ecology [1]–[3]. Analyses of plant species associations provide information about environmental heterogeneity, biotic interactions and patterns of seed dispersal [4]–[7]. This information is of particular interest in semi-arid plant communities where vegetation often occurs in patches. Usually, vegetation patches are composed of shrubs that can act as shelter against harsh environmental conditions. These shrubs are called ‘nurse plants’, for they appear to provide microhabitats that enhance survival for other plant species [2], [8]. Theoretical models based on empirical studies suggest that these positive interactions between plant species are one of the main drivers for the formation of these patches [8]–[10].

Since the article by Callaway and colleagues [11], additional studies have proliferated showing the presence and importance of positive interactions in plant communities [6], [7], [10]. It has been suggested that positive interactions should be particularly common in communities developing under high stress conditions and in those exposed to high consumer pressure [2], [11]. This theory has been referred to as the ‘Stress Gradient Hypothesis’ (SGH) and can explain some of the patterns in plant species interactions occurring in stressed ecosystems (but see [12], [13]). However, most studies have focused on a limited number of species within a community [14], and analyses at the community level have not provided unequivocal support for SGH [15]–[17]. Experiments at this scale are complicated because each species responds in a particular way to each stress, and, typically, those responses can change depending on the species’ ontogeny, habitat, and type of stress considered [17]–[19]. Usually, the response of a community is viewed as the net importance of positive and negative interactions in the community (*sensu*, the proportion of the total interactions in the community that are positive or negative [20]).

One alternative to studying interactions at the community level has been using spatial association between plant species as a surrogate for quantifying interactions [7], [15], [16]. This correlative approach assumes spatially associated species result from positive interactions, and species that are negatively associated are segregated by competitive interactions [7]. When interactions between species are weak, plant species will associate at random. Spatial association has been employed for studying plant species interactions in arid communities. For example, Verdú and Valiente-Banuet studied positive spatial associations between shrubs and seedlings in the Sonoran Desert, and found that there was a relationship between positive interactions and co-evolutionary processes in that plant community [21].

Grazing is one of the most important biotic factors shaping plant communities. Biomass consumption by herbivores affects both plant species composition and community spatial structure [22]–[25]. In arid and semi-arid ecosystems, grazing reduces total plant cover, increases abundance of certain life forms such as annual plants, and changes the identity of dominant species [24], [26]. Moreover, grazing may lead to increased positive interactions between plants as a result of associational defense; for example, some plant species protect themselves from herbivores by spatially associating with unpalatable plants [27]–[28]. Previous works testing SGH in ecosystems under grazing stress have found an increase in the importance of positive interactions at low grazing levels, but not at high grazing levels where negative interactions predominate [13], [29]. Assessing the effects of grazing on plant interactions provides valuable information for ecosystem management (e.g. which species act as refuge for grazing-sensitive species; which species need a refuge to survive). Changes in community structure are central to detecting when an ecosystem is overgrazed [24].

In the present work, we analyze the spatial associations among all plant species in a semi-arid community occurring along a gradient of livestock grazing. We evaluate associations for each individual species, between all pairs of species, and in the whole plant community, and how these associations change due to grazing. We estimate all associations by comparing real spatial co-occurrences between plant species with expected co-occurrences due to species abundances. Specifically, we hypothesize that the whole plant community will become more positively associated at intermediate grazing intensities, and that associations of each plant species and between pairs of species will depend on species life forms. We distinguish among associations between shrubs (those species responsible for patch formation), between grasses, and between shrubs and grasses.

## Materials and Methods

### Study Area and Data Collection

The study was conducted in Cabo de Gata-Níjar Natural Park, which lies along the Mediterranean coast in Southeastern Spain (36° 46′ N, 2° 09′ W). The park occupies 37,570-ha park, with a maximum elevation of 493 m (El Fraile Peak). The climate is semi-arid Mediterranean (marked seasonality, drought in summer and most rainfall in spring and autumn. Average annual rainfall = 193.9 mm, Average annual temperature = 19.4 C [30]). Historically, the area has been used as an agro-pastoral system, with cereal cropping on floodplains and livestock (sheep and goat) grazing on slopes all the year. The plant community is characterized by *Chamaerops humilis* L., and other common species include *Rhamnus lycioides* L., *Pistacia lentiscus* L., *Periploca laevigata* Aiton, and *Stipa tenacissima* L. [31].

Vegetation data were collected from the Southern section of the park, a volcanic area where highly stony soils predominate [32]. All permits required to carry out the field studies were obtained from the Natural Park authorities. In that region, vegetation is characterized by open shrubland with shrubs organized in patches, which are embedded in a matrix of a large tussock grass, *S. tenacissima* L. *S. tenacissima* is a very strong competitor that colonizes the gaps within patches caused by livestock and aridity, and can exclude other species once it becomes the dominant species [10], [33].

Within the area, four sites at different distances from El Romeral farm were selected. Movements of animals (sheep and goats) were monitored for one week per season with a GPS device. Effective stocking rate was calculated from the average stocking rate of the farm (ind·ha^−1^) multiplied by a correction factor based on the percentage of time each grazing site was used. Sites were ranked based on the amount of grazing pressure to which they had been exposed (G1 = 0 ind·ha^−1^; G2 = 0.27 ind·ha^−1^; G3 = 0.46 ind·ha^−1^; and G4 = 0.65 ind·ha^−1^). Grazing carrying capacity for this plant community is 0.39–0.57 ind·ha^−1^, so we considered G2 as low grazing intensity, G3 as intermediate grazing intensity and G4 as high grazing intensity [34]. In April, 2001, three 500-m-long linear transects were established at each site, and the Point-Intercept Method [35] was used to quantify vegetation. On each transect, the species occurring at each point were recorded every 20 cm. Presence and life form (shrub or grass) of all species were recorded and no distinction was made between the ontogenetic stages of individuals. All transects were run parallel to the slope, separated by 50 m and established at the same altitude, orientation and soil parent material.

### Association Measurements

A plant-plant association matrix *A_SxS_* was built for each site, based on the data pooled from the three transects. *S* is the number of species present at the site. In the matrix, *a_ij_* is the number of times that species *i* and *j* co-occurred at the same sampling point (with *a_ij_* = *a_ji_*). The matrix was used to calculate the total number of co-occurrences for a given species *i* (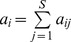) and the total number of co-occurrences at a site (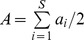). The diagonal terms of the matrix were set to 0 because it was not possible to estimate the co-occurrence of a species with itself from the presence data.

To test the deviation from the expected patterns of plant species associations, an *E_SxS_* matrix was calculated for each site. This matrix includes the expected number of co-occurrences between species based on their abundances. For each species *i*, its relative cover was calculated as *p_i_* = n_i_*T, where *n_i_* is the number of points where species *i* occurred at each site, and *T* is the total number of points sampled (2501 sampling points * 3 transects = 7503). In that context, *p_i_* is the probability of finding the species *i* at one randomly selected point at a site. Using those data for all of the plant species, a *P_SxS_* matrix was computed, where *p_ij_*  =  pi*pj. Therefore, *p_ij_* is the probability of finding the species *i* and *j* at the same sampling point on the site. The expected co-occurrences matrix *E_SxS_* was computed as *E_SxS_*  =  P_SxS_*T (with *e_ij_*  =  p_ij_*T), and total expected co-occurrences for each species *i* (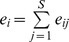) and each site (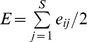) were calculated similarly as for *a_i_* and *A*.

A Poisson distribution was employed to compare *A_SxS_* and *E_SxS_*. The Poisson distribution is a statistical distribution widely used for analyzing count data. Furthermore, it has long been used in ecological analyses [36], particularly with vegetation data collected using the Point-Intercept Method (i.e. the number of contacts of a given species fits a Poisson distribution if individuals are distributed randomly and the probability of more than one contact for the same individual is negligible [35]). The Poisson distribution is characterized by one parameter, *λ*, which determines the mean and variance of the distribution. Thus, each value of *A*, *a_i_* and *a_ij_* was compared to a Poisson distribution fitted with its corresponding value, *E*, *e_i_* and *e_ij_*, as the *λ* parameter. To determine whether observed co-occurrences (*A*, *a_i_* and *a_ij_*) differed significantly from the co-occurrences expected based on species abundances (*E*, *e_i_* and *e_ij_*), 95% confidence intervals were calculated for each distribution. The comparison between *A* and *E* is the general association pattern present in the plant community (plant community presents more, less or the expected total number of co-occurrences). The comparison between *a_i_* and *e_i_* is the general type of association of species *i* (species *i* presents more, less or the expected total co-occurrences with all other species), while the comparison between *a_ij_* and *e_ij_* is the particular association between species *i* and *j* (species *i* co-occurs more, less or as expected with species *j*). Plant species were believed to be positively associated when they co-occurred at a level greater than that expected by chance. Negative associations between plants were inferred when co-occurrences were less frequent than expected by chance’. If actual co-occurrence values fell within the confidence interval, these values did not differ significantly from those expected because of species abundance and were considered a neutral association.

To assess possible changes in community associations along the grazing gradient, the importance of positive/negative associations at each site was measured as the relative increase/decrease in total co-occurrences with respect to expectations (*R*  =  (*A* - *E*)/*E*). The positive association dominates the plant community if *R* >0, whereas if *R* <0, the negative association is more dominant. If *R* ∼ 0, neither association dominates. Total co-occurrences (*A* and *E*) include all species in the plant community, but do not provide information about the number of species or pairs of species exhibiting each particular type of association. Therefore, we calculated the proportion of species and pairs of species that presented positive, negative and neutral associations. However, when species are uncommon it is not possible to detect a negative association because 0, the minimum observable value for *a_i_* and *a_ij_*, falls within the 95% confidence interval of the expected distribution. Therefore, we only considered species and pairs of species that were sufficiently abundant so that we could distinguish between positive, negative and neutral associations. For *a_i_*, the proportions of species considered at each site were G1 = 70%, G2 = 69%, G3 = 57%, and G4 = 64%; and for *a_ij_* the proportions of pairs of species considered were G1 = 3.3%, G2 = 4.1%, G3 = 3.3%, and G4 = 3%. Because it was possible to distinguish between neutral and positive associations among all species and pairs of species, we calculated the importance of positively associated species and pairs of species as the proportion of positive associations in relation to all possible associations (species, *Rs*  =  *s*+/*S*; and pairs of species, *Rss*  =  *ss*+/*S*(*S* - 1); where *s*+ is the number of species showing a positive association, and *ss*+ is the number of pairs of species that are positively associated with each other). As *Rs* and *Rss* increase, more plant species represent a positive association, and more pairs of species are positively associated. *Rs* was calculated for both life forms and for all species, and *Rss* was calculated for associations between species with the same life form, between different life forms and between all species. All analyses were performed using R (http://www.R-project.org). The variables and parameters used in the analyses are presented in table 1.

**Table 1:** Variable and parameter codes employed in the study.

Code	Description
*T*	Total number of surveyed points
*S*	Number of species recorded
*n_i_*	Abundance of species *i*
*p_i_*	Relative abundance of species *i*
*a_ij_*	Co-occurrences between species *i* and *j*
*a_i_*	Total co-occurrences of species *i*
*A*	Total co-occurrences at the site
*A_SxS_*	Matrix with *a_ij_* values
*p_ij_*	Co-occurrence probability of species *i* and *j*
*P_SxS_*	Matrix with *p_ij_* values
*e_ij_*	Expected co-occurrences between species *i* and *j*
*e_i_*	Expected total co-occurrences of species *i*
*E*	Expected total co-occurrences
*E_SxS_*	Matrix with *e_ij_* values
*s*+	Species with positive associations*
*ss*+	Pairs of species with positive associations*
*R*	Deviation from total co-occurrences at the site
*Rs*	Proportion of species with positive associations at the site*
*Rss*	Proportion of pairs of species with positive associations at the site*

Each value was calculated for each of four sampling sites within the study area.

* When *a_i_* and *a_ij_* are higher than *e_i_* and *e_ij_*, and fall out of their confidence intervals.

## Results

Grazing modified the plant community in Cabo de Gata-Níjar Natural Park. Species richness was 70% greater at the ungrazed than grazed sites, particularly due to the large number of grass species, and biodiversity decreased as grazing became more intense (Table 2). The mean number of species recorded per point was largest at G1 and the number of points with no records (Bare soil) was largest at low and intermediate grazing values (G2 and G3). Mean number of co-occurrences per point decreased with grazing. Abundance (*n_i_*) and co-occurrences (*a_i_*) of each species for each grazing intensity are included as support information (Table S1).

**Figure 1:**
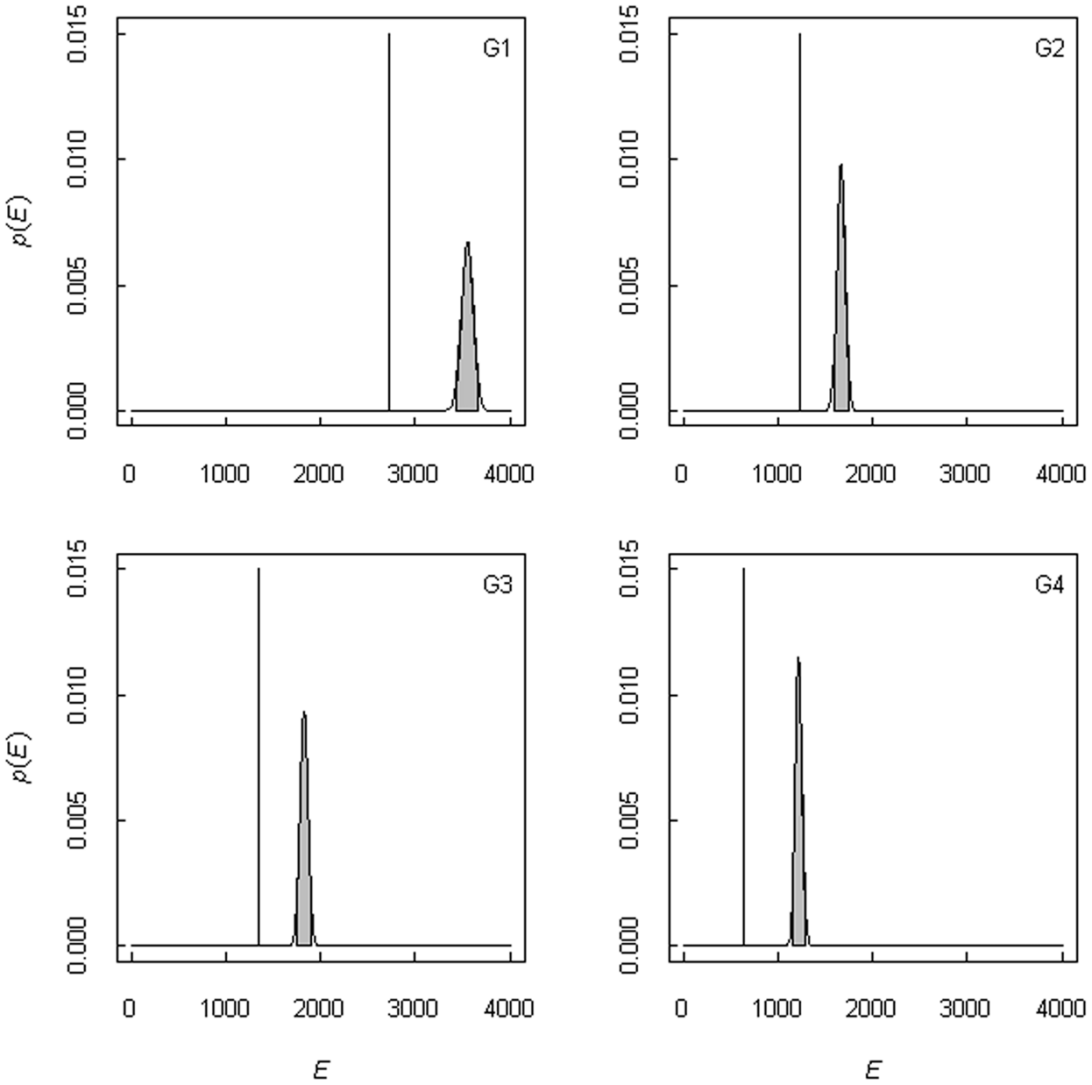
Distribution of expected total co-occurrences for the entire plant community in Cabo de Gata-Níjar. *E*, plant community total co-occurrences; *p*(*E*), probability distribution of expected total co-occurrences *p*(*N*/*E*) = (1/*N*!)*e^-A^*A*N, where *N* is the number of surveyed points (7503). G1 to G4 are the sampling sites and are sorted by grazing intensity (G1 = 0 ind·ha^−1^; G2 = 0.27 ind·ha^−1^; G3 = 0.46 ind·ha^−1^; and G4 = 0.65 ind·ha^−1^). Vertical lines represent *A*. Because the observed values are smaller and fall outside the 95% confidence interval (grey area), the community is considered to be dominated by negative associations.

At all four sampling sites, plant communities exhibited fewer total co-occurrences (*A*) than were expected by chance (*E*), which indicated that plants were more likely to be alone in this community, rather than in association (Fig. 1). Negative association was most important at the highest grazing intensity (*R_G1_* = −0.233, *R_G2_* = −0.262, *R_G3_* = −0.269, *R_G4_* = −0.476).

**Table 2:** Characteristics of study sites in Cabo de Gata-Níjar Natural Park.

Site	Latitude	Longitude	BS	*S*	*S*/point	Ev	*A*/point
G1	36° 46′ 6″ N	2° 10′ 37″ W	2050	119 (24/95)	1.03	0.645	0.363
G2	36° 44′ 45″ N	2° 8′ 45″ W	2982	70 (22/48)	0.75	0.559	0.163
G3	36° 45′ 24″ N	2° 8′ 12″ W	2750	74 (26/48)	0.787	0.515	0.177
G4	36° 45′ 5″ N	2° 7′ 52″ W	2360	72 (18/56)	0.764	0.39	0.084

BS, Bare soil, number of points with no species; *S*, species richness (Shrub species/Grass species); *S*/point, mean number of species at each point; Ev, evenness (Shannon diversity/log(S)); *A*/point, mean number of co-occurrences per point (*A*/7503).

**Figure 2:**
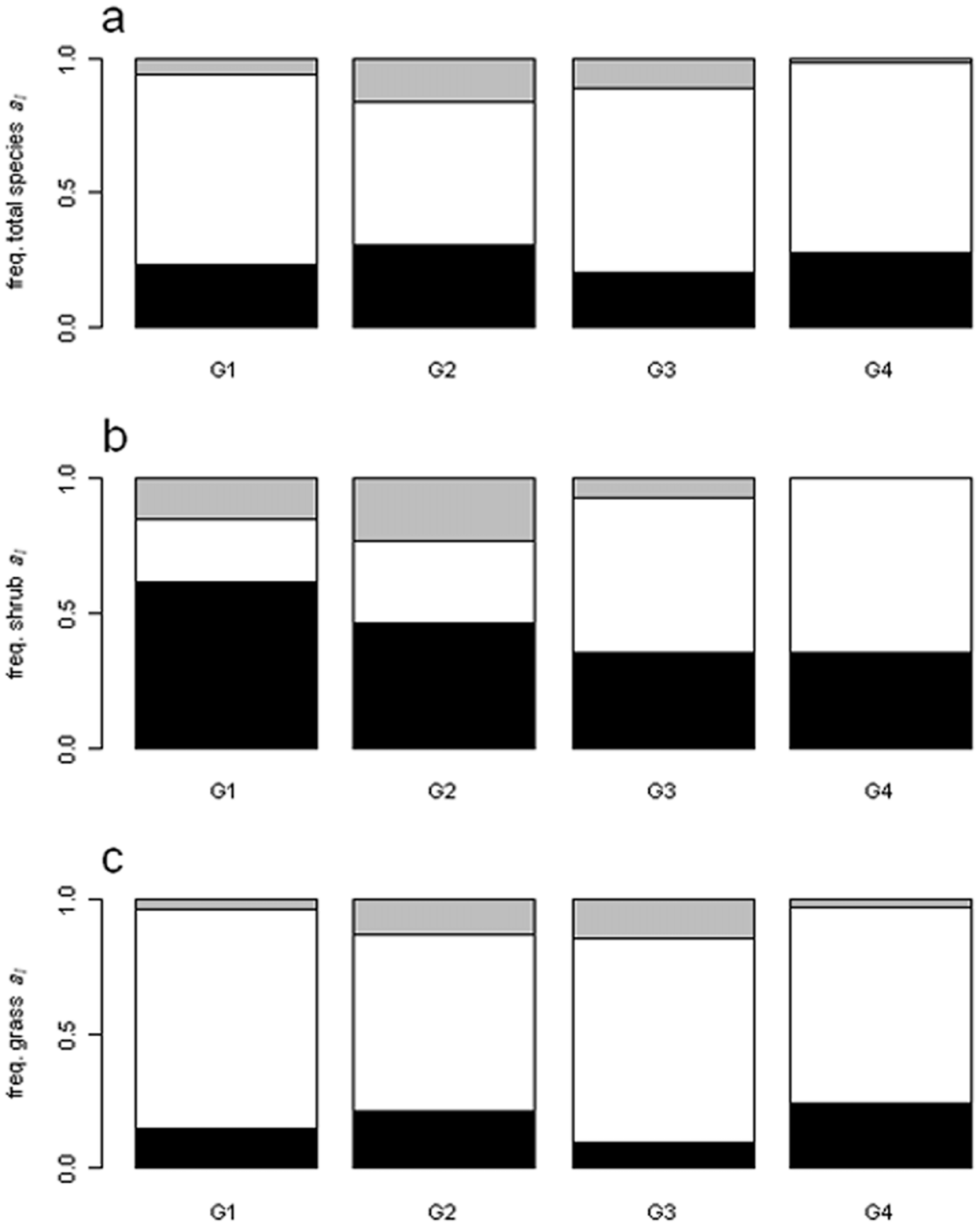
Relative number of species for each type of association. a, proportion of species with each type of association; b,proportion of shrubs with each type of association; c, proportion of grasses with each type of association; G1 to G4 are the sampling sites. Associations are classified as negative (black area), neutral (white area) or positive (grey area) depending on the relationship between *a_i_* value and the expected distribution. Only species that could allow distinguishing negative from neutral associations were employed.

**Figure 3:**
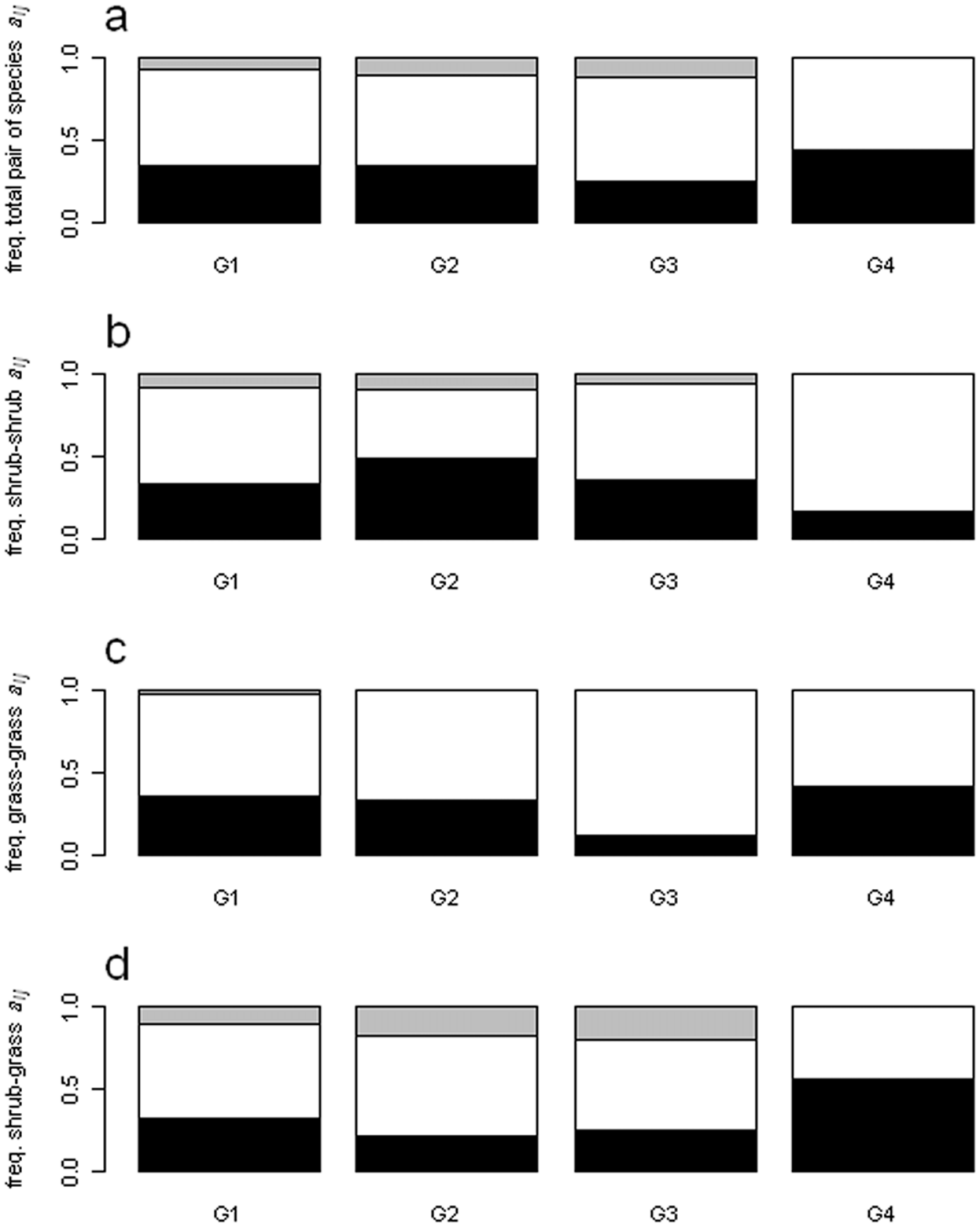
Relative number of pairs of species for each type of association. a, proportion of pairs of species with each type of association; b, proportion of types of association between pairs of shrubs; c, proportion of types of association between pairs of grasses; d, proportion of types of association between pairs of shrubs and grasses. G1 to G4 are the sampling sites. Associations are classified as negative (black area), neutral (white area) or positive (grey area) depending on the relation between *a_ij_* value and the expected distribution. Only pairs of species that could allow distinguishing negative associations were employed.

Regarding the general type of association of species (*a_i_*), at all sampling sites there were species which associated positively, neutrally or negatively, but species that associated neutrally were the most common. Besides neutrally associated species, those negatively associated were more frequent than positively associated ones (Fig. 2a). No species presented a shift in its type of association along the grazing gradient (i.e. no species exhibited both a positive and a negative type of association along the gradient, Table 3). Negatively associated shrubs were common, particularly at the non-grazed site (Fig. 2b), while negatively associated grasses increased at sites with highest grazing (Fig. 2c). For associations between pairs of species (*a_ij_*), all three types of association were also detected. Neutral associations were the most common, and negative associations were more common than positive ones (Fig. 3a). Negative associations between shrubs were highest in areas with low and intermediate grazing intensities (Fig. 3b), whereas negative associations between grasses and between shrubs and grasses were highest at high grazing intensity (Fig. 3c–d). Individually, each species could associate positively with some species, and neutrally or negatively with the rest.

**Table 3:** Plant species association type along a grazing gradient in Cabo de Gata-Níjar.

Life form	Species	G1	G2	G3	G4
Shrubs	*Anthyllis cytisoides* L.	0	+	0	0
	*Ballota hirsuta* Benth	0	+	0	0
	*Chamaerops humilis* * L.	−	−	−	−
	*Dianthus charidemi* Pau.	0	0	+	0
	*Genista ramosissima* * (Desf.) Poir.	−	−	−	0
	*Genista spartoides* Spach	−	0	0	0
	*Genista umbellata* * Poiret	0	0	−	0
	*Launaea lanifera* * Pau.	−	−	−	−
	*Lavandula multifida* * L.	0	0	−	0
	*Lycium intrincatum* Boiss	0	0	0	−
	*Periploca laevigata* * Aiton.	−	−	0	0
	*Phagnalon saxatile* L.	0	+	0	0
	*Phlomis lychnitis* L.	−	0	0	0
	*Phlomis purpurea* * L.	+	0	0	0
	*Salsola genistoides* * Juss. ex Poiret	−	−	0	−
	*Teucrium charidemi* * Sandw.	+	0	0	0
	*Thymus hyemalis* * Lange	−	−	−	−
Grasses	*Asparagus albus* L.	0	+	+	0
	*Asphodelus* sp. L.	−	0	0	0
	*Asphodelus tenuifolius* * Cav.	0	0	0	−
	*Avena sterilis* sp. L.	0	−	0	0
	*Brachypodium distachyon* * L.	0	0	0	−
	*Brachypodium retussum* * (Pers.) Beauv	0	+	+	+
	*Euphorbia segetalis* L.	0	+	+	0
	*Hippocrepis ciliata* Willd.	−	0	0	0
	*Leontodon longirrostris* (Finch and Sell) Talavera.	0	−	0	0
	*Lygeum spartium* * L.	−	0	0	0
	*Medicago truncatula* Gaertner	0	0	0	−
	*Melica minuta* * L.	0	+	0	0
	*Plantago afra* L.	0	−	0	−
	*Plantago albican* L.	0	−	0	0
	*Plantago amplexicaulis* Cav.	0	0	0	−
	*Plantago bellardi* * Ail.	−	0	−	−
	*Reichardia picroides* (L.) Roth	+	0	0	0
	*Sonchus tenerrimus* L.	+	0	0	0
	*Stipa capensis* Thunb	−	0	0	0
	*Stipa tenacissima* * L.	−	−	−	−
	*Viola arborescens* L.	0	−	0	0

G1-4, study sampling sites; +, *a_i_* > *e_i_*; −, *a_i_* < *e_i_* (*a_i_* must fall outside the 95% confidence interval of *e_i_*).

* . species that had *p_i_* >0.01. Only those species that differed significantly from expectation on at least one site were included in the table.

**Figure 4:**
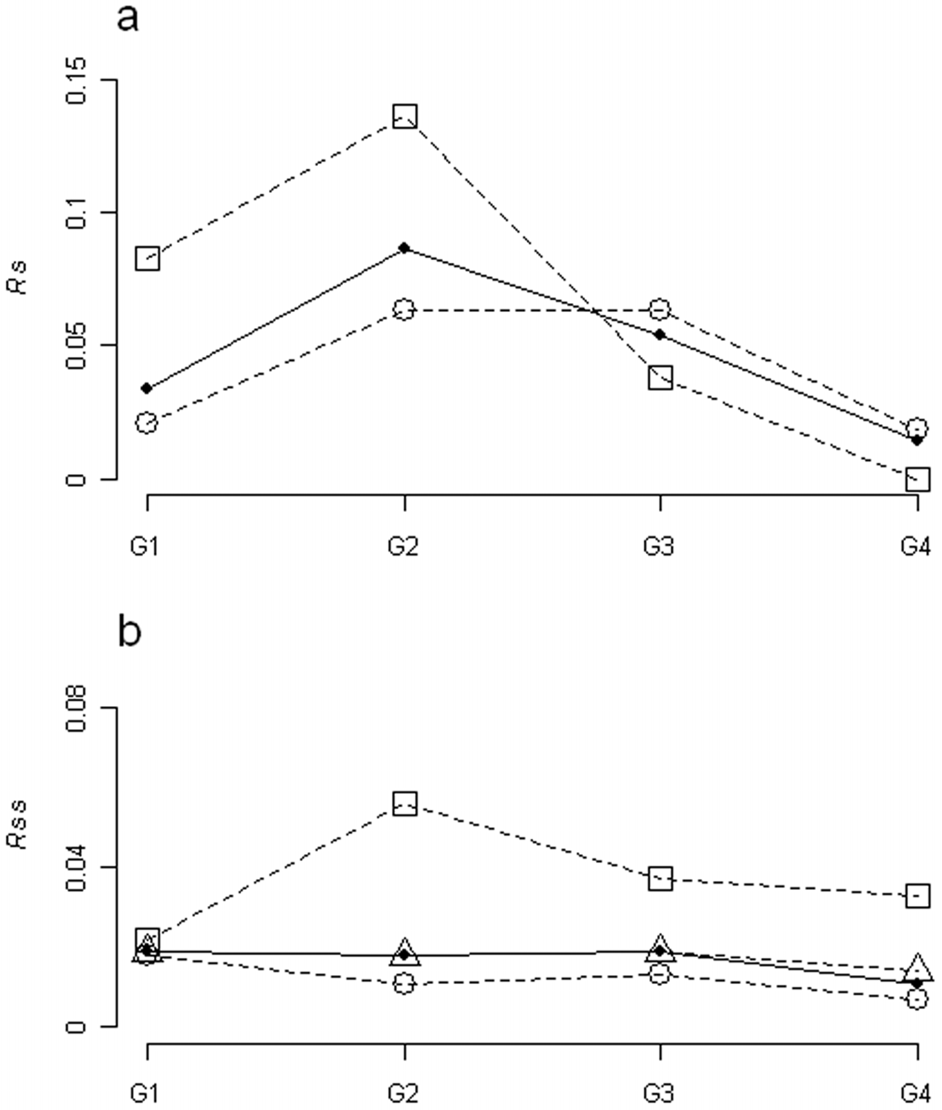
Importance of positive associations along grazing gradient. a, *Rs*, importance of positively associated species for each life form. b, *Rss*, positively associated pairs of species importance for each life form. G1 to G4 are the sampling sites. In a, importance of positive associations for species is evaluated for shrubs (open circles), grasses (open squares) and all species (black circles). In b, importance of positive associations for pairs of species is evaluated for shrub-shrub (open circles), grass-grass (open squares), shrub-grass (open triangles) and for all associations of pairs of species (black circles).

The importance of species with positive association (*Rs*) was highest at low grazing levels for both life forms and for all species (Fig. 4a). The importance of positive associations between all pairs of species (*Rss*) remained nearly constant, but they decreased at the site with the highest level of grazing (Fig. 4b). The importance of positive associations between shrubs was highest at low grazing intensities, whereas between grasses and between shrubs and grasses it was highest at the non-grazed site.

## Discussion

In Cabo de Gata-Níjar Natural Park, plant community responded to grazing intensity. Grazing reduced community biodiversity, and increased bare soil except at the site with highest grazing intensity (Table 2). Grazers preferentially feed on palatable species, favoring the persistence of non-palatable species [24]. This selective removal modifies the abundance of plant species, with some species becoming dominant while others disappear from the community [26]. The effect of grazing was particularly remarkable for grasses, which lost half their species. When grazing became very intense, the plant community was dominated by one very abundant species (*S. tenacissima*). These changes in species composition and abundance also modified the associations between species in the plant community.

Regarding the general association pattern in the community, there were fewer co-occurrences than expected by chance. At all sites, irrespective of grazing intensity, plants showed a tendency to segregate rather than associate. Thus, it seemed that negative associations dominated the community, particularly at maximum grazing intensity. In recent works in the Spanish Mediterranean region, positive interactions between plant species have been presented as a main determinant of the plant community [37]. Here the predominant interaction across all grazing intensities was negative. Furthermore, associations among abundant species were predominantly negative. Grasses such as *S. tenacissima*, and shrubs like *Launaea lanifera* and *Thymus hyemalis* generally associated negatively with other species independently of grazing level. These species are common in degraded habitats and, through competitive exclusion (*S. tenacissima* [33]) or allelopathy (*T. hyemalis* [38]), they usually occur alone in the area rather than in association. In this area, abundant plant species are adapted to the harsh semi-arid environment and grazing, and they compete with other well-adapted species for space and other resources [39]. On the other hand, some abundant species benefit from the association with other plant species. For example, the grass *Brachypodium retussum* preferentially develops under the canopy of other species [33] and the shrub *Phlomis purpurea* presents low-density branching, which allows it to enter vegetation patches [40]. Although some plant taxa exhibited positive associational patterns, neutral and negative associational patterns were most common across all species in the communities.

Large shrubs such as *Chamaerops humilis*, *Genista ramosissima* and *Periploca laevigata* are responsible for the development of vegetation patches in the area [31]. Often, in semi-arid environments, these shrub species act as ‘nurse plants’ because they facilitate establishment and development of species under their canopy [21], [41], [42]. In our study, several positive associations between shrubs or between shrubs and grasses reflect this nursery effect. However, negative associations between these shrubs and the competitive abundant species overcame the positive association that shrubs established with other species. There are some examples of the facilitative effect of grasses on the establishment of other species in semi-arid ecosystems [43], but in our case most of the positive associations included at least one shrub, suggesting the role shrubs have as ‘nurse plants’ in the community.

In order to analyze associations of species and pairs of species, we considered only those species abundant enough to allow distinguishing negative from neutral associations. Typically, in plant community studies uncommon species are excluded from the analyses because they do not provide robust results [7], [44]. In our case, despite the low likelihood of detecting negative associations in uncommon species, we found many positive associations between these species. Others have suggested that rare species are more likely to be facilitated than abundant ones [44]. Our results also suggest that rare species are likely to be associated with other species in semi-arid plant communities.

In our study, the proportion of positive species associations increased at low and intermediate grazing intensities (G2–3) and was lowest at the highest intensity (G4). This result has been reported in other studies dealing with changes in the interactions between plant species along grazing gradients, but to our knowledge this is the first time that this result is evaluated at community level [13], [26], [29]. As grazing increases, associations between plant species become more frequent, particularly those associations with shrub species that act as refuges against grazers. However, once a particular threshold is reached, grazing intensity is such that facilitative species cannot provide protection and positive associations decrease [29]. Interestingly, the particular type of association of each species remained consistent across the grazing gradient. One possible explanation is that each species exhibits a predominant associative type in the community, regardless of grazing intensity (i.e., competitive species never become associative [25]). Others have documented associational changes across different stages of plant ontogeny [19], but here the observed associations reflect the general type of association of the species in the area.

Spatial associations between species have been presented as an indirect measure of species interactions, but this approach has limitations. The spatial association of species is the net result of biotic interactions, seed dispersal and environmental heterogeneity [45]. The present study has additional limitations. For example, inter-specific associations are not measured, all individuals of each species are considered ecologically identical (e.g. different life stages may interact differently [19], [46]) and other effects of interactions are ignored (e.g changes in species biomass [14]). Nevertheless, our results are consistent with those reported by works about SGH including a limited number of species [13], [27]. Identifying the processes that drive the organization of natural communities under grazing and the role that each species plays in said organization provides valuable information about which species maintain the structure of grazed ecosystems. This information is central to detect overgrazing events in grazed ecosystems [24].

In conclusion, most species were not associated with other species and the most common association among plants in this semi-arid plant community was negative, especially associations with dominant species. This suggests that either neutral processes and/or competitive interactions are structuring these plant communities. The associational patterns of most species did not vary with grazing intensity; however, there was a tendency for positive associations among species to become less frequent at high levels of grazing. Positive associations among plants appeared to be most important at low and intermediate grazing intensities. Identifying non-neutral species associations provide information about the processes and species driving the organization of natural communities and helps further the development of conservation and restoration plans.

## Supporting Information

Table S1 **Life form, abundance (** ***n_i_*** **), co-occurrences (** ***a_i_*** **) and association type (** ***ass*** **) of plant species in Cabo de Gata-Níjar Natural Park along grazing gradient.** Association values are presented for species that could distinguish between neutral and negative associations.
(XLSX)

## References

[1] Ripley BD (1977). Modelling spatial patterns.. J Roy Stat Soc B Met.

[2] Bertness MD, Callaway RM (1994). Positive interactions in communities.. Trends Ecol Evol.

[3] Maestre FT, Rodríguez F, Bautista S, Cortina J, Bellot J (2005). Spatial associations and patterns of perennial vegetation in a semi-arid steppe: a multivariate geostatistics approach.. Plant Ecol.

[4] Casper BB (1996). Demographic consequences of drought in the herbaceous perennial cryptantha flava: Effects of density, associations with shrubs, and plant size.. Oecologia.

[5] Rees M, Grubb PJ, Kelly D (1996). Quantifying the impacts of competition and spatial heterogeneity on the structure and dynamics of a four-species guild of winter annuals.. Am Nat.

[6] Escudero A, Romao RL, de la Cruz M, Maestre FT (2005). Spatial pattern and neighbour effects on Helianthemum squamatum seedlings in a Mediterranean gypsum community.. J Veg Sci.

[7] Tirado R, Pugnaire F (2005). Community structure and positive interactions in constraining environments.. Oikos.

[8] Pugnaire FI, Haase P, Puigdefabregas J (1996). Facilitation between higher plant species in a semiarid environment.. Ecology.

[9] Aguiar MR, Sala OE (1999). Patch structure, dynamics and implications for the functioning of arid ecosystems.. Trends Ecol Evol.

[10] Alados CL, Gotor P, Ballester P, Navas D, Escós JM (2006). Association between competition and facilitation processes and vegetation spatial patterns in alpha steppes.. Biol J Linn Soc.

[11] Callaway RM, DeLucia EH, Moore D, Nowak R, Schlesinger WH (1996). Competition and facilitation: Contrasting effects of Artemisia tridentata on desert vs. montane pines.. Ecology.

[12] Maestre FT, Cortina J (2004). Do positive interactions increase with abiotic stress? a test from a semi-arid steppe.. P Roy Soc Lond B Bio.

[13] Smit C, Rietkerk M, Wassen MJ (2009). Inclusion of biotic stress (consumer pressure) alters predictions from the stress gradient hypothesis.. J Ecol.

[14] Brooker RW, Maestre FT, Callaway RM, Lortie CL, Cavieres LA (2008). Facilitation in plant communities: The past, the present, and the future.. J Ecol.

[15] Cavieres LA, Arroyo MTK, Penaloza A, Molina-Montenegro MA, Torres C (2002). Nurse effect of Bolax gummifera cushion plants in the alpine vegetation of the Chilean Patagonian Andes.. J Veg Sci.

[16] Cavieres LA, Badano EI, Sierra-Almeida A, Gómez-Gónzalez S, Molina-Montenegro MA (2006). Positive interactions between alpine plant species and the nurse cushion plant Laretia acaulis do not increase with elevation in the Andes of central Chile.. New Phytol.

[17] Engel EC, Weltzin JF (2008). Can community composition be predicted from pairwise species interactions?. Plant Ecol.

[18] Tielborger K, Kadmon R (2000). Temporal environmental variation tips the balance between facilitation and interference in desert plants.. Ecology.

[19] Miriti MN (2006). Ontogenetic shift from facilitation to competition in a desert shrub.. J Ecol.

[20] Brooker RW, Kikvidze Z, Pugnaire FI, Callaway RM, Choler P (2005). The importance of importance.. Oikos.

[21] Verdú M, Valiente-Banuet A (2008). The nested assembly of plant facilitation networks prevents species extinctions.. Am Nat.

[22] Belsky AJ (1986). Does herbivory benefit plants? a review of the evidence.. Am Nat.

[23] McNaughton SJ (1986). On plants and herbivores.. Am Nat.

[24] Milchunas DG, Lauenroth WK (1993). Quantitative effects of grazing on vegetation and soils over a global range of environments.. Ecol Monogr.

[25] Olff H, Ritchie ME (1998). Effects of herbivores on grassland plant diversity.. Trends Ecol Evol.

[26] Bisigato AJ, Bertiller MB (1997). Grazing effects on patchy dryland vegetation in northern Patagonia.. J Arid Environ.

[27] Graff P, Aguiar MR, Chaneton EJ (2007). Shifts in positive and negative plant interactions along a grazing intensity gradient.. Ecology.

[28] Baraza E, Zamora R, Hodar JA (2006). Conditional outcomes in plant-herbivore interactions: neighbours matters.. Oikos.

[29] Smit C, Vandenberghe C, den Ouden E, Muller-Scharer H (2007). Nurse plants, tree saplings and grazing pressure: changes in facilitation along a biotic environmental gradient.. Oecologia.

[30] Passera C (1999). Propuestas metodologicas para la gestion de ambientes forrajeros naturales de zonas aridas y semiaridas.. Ph.D. thesis, Universidad de Granada, Granada.

[31] Peinado M, Alcaraz F, Martínez-Parras JM (1992). Vegetation of southeastern Spain, volume X of Flora et Vegetatio Mundi.. Berlin: J. Cramer.

[32] Oyonarte C, Pérez-Pujalte A, Gil C, Sánchez G (1999). Cartografía y delimitación de unidades geomorfoedáficas en el Parque Natural de Cabo de Gata-Nijar.. Sevilla: Junta de Andalucía.

[33] Alados CL, Pueyo Y, Giner ML, Navarro T, Escós J (2003). Quantitative characterization of the regressive ecological succession by fractal analysis of plant spatial patterns.. Ecol Model.

[34] Robles AB, Passera CB (1995). Native forage shrub-species in south-eastern Spain: forage species, forage phytomass, nutritive value and carrying capacity.. J Arid Environ.

[35] Goodall DW (1952). Some considerations in the use of point quadrats for the analysis of vegetation.. Aust J Biol Sci.

[36] Holt AR, Gaston KJ, He F (2002). Occupancy-abundance relationships and spatial distribution: a review.. Basic Appl Ecol.

[37] Pugnaire F, Armas C, Maestre FT (2011). Positive plants interactions in the Iberian Southeast: Mechanisms, environmental gradients, and ecosystem function.. J Arid Environ.

[38] Pugnaire F, Armas C, Valladares F (2004). Soil as a mediator in plant-plant interactions in a semi-arid community.. J Veg Sci.

[39] Fowler N (1986). The role of competition in plant communities in arid and semiarid regions.. Annu Rev Ecol Syst.

[40] Navarro T, Pascual V, Cabezudo B, Alados CL (2009). Architecture and functional traits of semi-arid shrub species in Cabo de Gata Natural Park, SE Spain.. Candollea.

[41] Verdú M, Valiente-Banuet A (2011). The relative contribution of abundance and phylogeny to the structure of plant facilitation networks.. Oikos.

[42] Niering WA, Whittaker RH, Lowe CH (1963). The saguaro: a population in relation to environment.. Science.

[43] Maestre FT, Bautista S, Cortina J, Bellot J (2001). Potential for using facilitation by grasses to establish shrubs on a semiarid degrade steppe.. Ecol Appl.

[44] Choler P, Michalet R, Callaway RM (2001). Facilitation and competition on gradients in alpine plant communities.. Ecology.

[45] Barot S, Gignoux J, Menaut JC (1999). Demography of a savanna palm tree: predictions from comprehensive spatial pattern analyses.. Ecology.

[46] McAuliffe JR (1988). Markovian dynamics of simple and complex desert plant communities.. Am Nat.

