# Correction: Changes in Semi-Arid Plant Species Associations along a Livestock Grazing Gradient

**DOI:** 10.1371/journal.pone.0091478

**Published:** 2014-02-27

**Authors:** The PLOS ONE Staff

There is an error in the calculation of p(i). In both the PDF and online versions of the article, p(i) is calculated as n(i)*T, which is incorrect. Instead, p(i) should be calculated as n(i)/T.
